# Leveraging the “mad genius” debate: why we need a neuroscience of creativity and psychopathology

**DOI:** 10.3389/fnhum.2014.00771

**Published:** 2014-09-29

**Authors:** Shelley Carson

**Affiliations:** Department of Psychology, Harvard UniversityCambridge, MA, USA

**Keywords:** cognitive disinhibition, creativity, latent inhibition, neuroscience, psychopathology, shared vulnerability model

In this opinion article, I summarize the so-called “mad genius” debate and suggest a way to reframe the issue so that it can benefit the field rather than divide it. As others have pointed out, creativity cannot effectively be studied as an overarching entity; it must be broken into smaller pieces and studied from an individual differences perspective in order to provide meaningful results relating to brain function. I also try to frame the infant field of creativity-psychopathology neuroscience, and I end with the benefits this area of inquiry will provide.

## The “mad genius” debate

Creativity has been described as a human survival mechanism that allows both the individual and the species to adapt to the environment in real time (Richards, 1999; Miller, 2001). Creativity is also a sought-after character trait in fields as diverse as business, the arts, science, and sports. And yet we find there is a long list of high-level creative achievers who have suffered from the inner demons of psychopathology. The list includes such contemporary luminaries as comedian Robin Williams, as well as a host of influential creators from the past: Vincent Van Gogh, Robert Schumann, Mozart, Beethoven, Sylvia Plath, Virginia Woolf, Anne Sexton, Ernest Hemingway, Edgar Allan Poe, Michelangelo, Georgia O'Keefe, and Jackson Pollock, to name only a few (Jamison, 1993). Mentions of a connection between creativity and madness extend back at least as far as Aristotle and Plato (Becker, 2000-2001). These anecdotal examples appear to contradict the beliefs that creativity is adaptive for the individual and is, as suggested by some (e.g., Maslow, 1968; Dietrich, 2014), a manifestation of positive mental health.

In addition to anecdotal examples, we see a growing body of empirical studies associating creativity with various forms of psychopathology, including mood disorders (for reviews, see Johnson et al., 2012; Kaufmann and Kaufmann, 2014), schizotypal thinking (for a review, see Barrantes-Vidal, 2014), alcohol abuse (Andreasen, 1987; Dardis, 1989; Ludwig, 1990, 1992; Post, 1994), and more recently ADHD (Healey and Rucklidge, 2006; Healey, 2014) and autism (Pring et al., 2012). These studies appear to indicate that highly creative individuals are at greater risk for certain disorders than are members of the general public. However, more nuanced research suggests that individuals with *small* doses of psychopathology, such as those who exhibit low-level symptoms or who have inherited part—but not all—of a pathological genotype, are more likely to be creative than either their mentally healthy counterparts or those with full-blown disorder (Heston, 1966; Karlsson, 1970; Richards et al., 1988; Kinney et al., 2000-2001; Abraham, 2014; Simonton, 2014). This is often referred to as the “inverted U” model (Richards et al., 1988), or what I call a “dose-dependent” relationship (Carson, 2013), of creativity and psychopathology.

Many (perhaps most) of these studies which have found an association between creativity and psychopathology have been criticized for methodological deficits which question their validity (see Schlesinger, 2009; Sawyer, 2012; Dietrich, 2014). And so we have a “mad genius” debate, with one side suggesting that elements of mental illness may enhance creativity (at least in small doses) and the other side suggesting that the correlation between creativity and psychopathology is unsupported and that virtually all the studies that claim a connection are riddled with methodological errors.

## Reframing the debate

This debate, while divisive, may lead to important advances in the neuroscience of creativity because it calls attention to two problems that need to be addressed: first the presence (flawed studies not withstanding) of a host of creative individuals with psychopathology, and second, the need for methodological rigor when investigating the creativity/psychopathology connection. The World Health Organization estimates that 450 million people world-wide suffer from mental disorders (World Health Organization, 2013). Even if rates of psychopathology are actually *lower* among highly creative individuals than in the general population (and we don't currently have a body of research that corroborates this), it is still the case that there are a great many individuals (likely millions) who are both creative and who have mental disorders. The question within the field of neuroscience then should not be *whether* creative individuals are at greater risk for madness than the general population; it should be whether creativity and the creative process are *different* in the disordered brain than in the non-disordered brain.

Given the importance of creative thought to human survival, it is likely that throughout the course of evolution we have developed a variety of biologically-based strategies to help us solve ill-defined problems creatively (see Jung, 2014). What we refer to as “creativity” is actually a collection of these strategies rather than a single process or entity. Several of these strategies include visualizing outcomes of an action (mental imagery), generating multiple (both original and mundane) solutions to a prompt (divergent thinking), consciously making comparisons of between two disparate concepts or objects (metaphorical thinking), and putting aside a problem to allow it to incubate until a solution suddenly arrives (insight). Each of these strategies can be further broken down into component processes, some of which utilize brain networks that are already understood in terms of their underlying neuroscience. For example, we know that mental imagery utilizes much of the same circuitry that is used to process ordinary vision (Kosslyn et al., 2006). By continuing to study creativity as if it were a single entity, and by expecting that all subjects will engage similar neural circuits to solve creative tasks, we will only continue to generate conflicting and inconsistent findings (Arden et al., 2010; Dietrich and Kanso, 2010). However, by (1) parsing creativity into smaller components based on cognitive processes (e.g., Abraham, 2013), and (2) looking at neural creative processes through the lens of an individual differences approach (including differences in predisposition for psychopathology) we can eliminate much of the confusion and contradictory results in the field.

## A neuroscience of creativity-psychopathology

Individuals with a predisposition to mental disorder may utilize different strategies, or they may use familiar strategies in unusual ways, to solve creative tasks. For over a century, knowledge of psychopathological states in the brain has illuminated our knowledge of normal brain states, and that should also be the case with the study of the creative brain. Neuroscience can approach this study in two ways. First, it can identify genetic variations that may underlie both creativity and psychopathology. This molecular biology approach is already underway, with several studies indicating polymorphisms of the DRD2 and DRD4 genes (Reuter et al., 2006; Mayseless et al., 2013), the 5HT2a gene (Ott et al., 2005) and the NRG1 gene (Kéri, 2009) that have been associated with both creativity and certain forms of psychopathology.

Second, brain imaging work can be applied to the study of the cognitive mechanisms that may be commonly shared between creativity and psychopathology. For example, psychologists have long suggested that both schizotypal and highly creative individuals tend to utilize states of cognitive disinhibition to access associations that are ordinarily hidden from conscious awareness (e.g., Kris, 1952; Koestler, 1964; Eysenck, 1995). Research is revealing that indeed both highly creative subjects and subjects who are high in schizotypy demonstrate more disinhibition during creative tasks than less creative or less schizotypal subjects (see Martindale, 1999; Carson et al., 2003; Abraham and Windmann, 2008; Dorfman et al., 2008). However, the neural substrates of cognitive disinhibition, as applied to creativity, need to be further studied.

My colleagues and I have found that cognitive disinhibition (in the form of reduced latent inhibition) combined with very high IQ levels predicts extraordinary creative achievement (Carson et al., 2003). These results have since been replicated (Kéri, 2011). We hypothesized that cognitive disinhibition allows a broadening of stimuli available to consciousness while high IQ affords the cognitive resources to process and manipulate that increased stimuli to form novel and creative ideas without the individual becoming overwhelmed and confused. What we did not test is whether the high creative achievers in our studies exhibited phasic changes in latent inhibition, or whether their reduced inhibition was more trait-like, as is seen in persons at risk for psychosis. Because latent inhibition tasks are compatible with neuroimaging, the study of controlled cognitive disinhibition is one area of potential study for the neuroscience of creativity.

**Figure 1:**
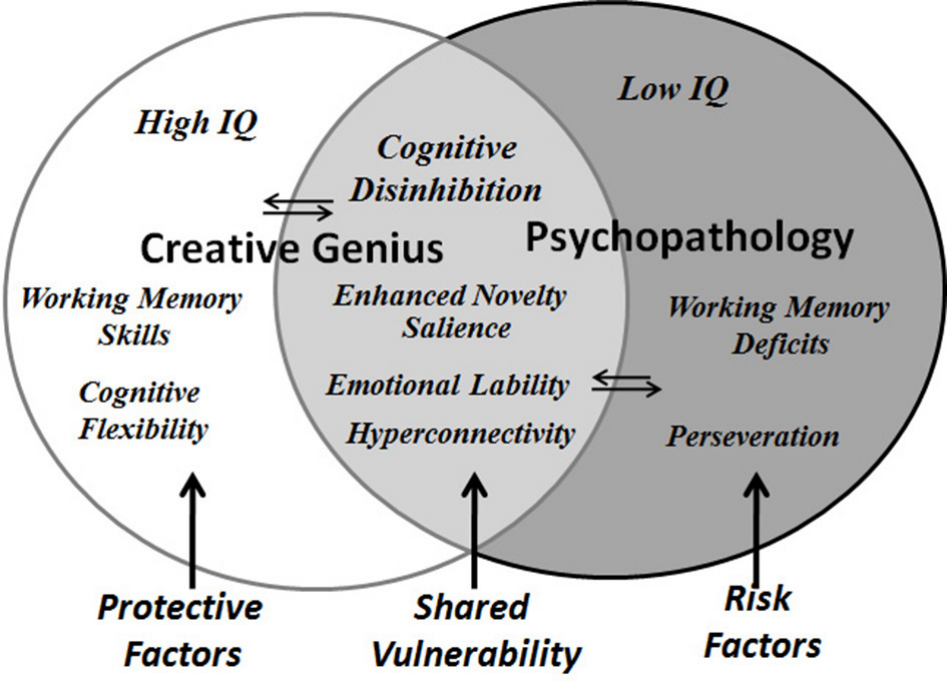
**Shared vulnerability model of creativity and psychopathology (adapted from Carson, 2011)**.

Additional areas of study are suggested by the shared vulnerability model of creativity and psychopathology (Carson, 2011, 2013). The shared vulnerability model suggests that creativity and psychopathology may share genetically-influenced factors that are expressed as either pathology or creativity depending upon the presence or absence of other moderating factors (see Figure 1). The shared vulnerability components that have been identified, in addition to cognitive disinhibition, include novelty salience, neural hyperconnectivity, and emotional lability.

Novelty salience is associated with the motivation to explore novel aspects of ideas or objects via the dopamine reward system. Novelty-seeking is associated with creative personality (McCrae, 1993; Reuter et al., 1995), creative drive (Flaherty, 2005), alcohol abuse and addiction (Frye and Salloum, 2006; Grucza et al., 2006), and with bipolar states of hypomania and mania (Minassian et al., 2011). Brain imaging studies can determine whether reward areas (such as the ventral striatum) are more active during creative tasks in individuals who score high (rather than low) on measures of creative achievement as well as in subjects who score high on scales of hypomania. Brain imaging studies can also investigate whether activation of reward areas and other neural networks varies during creative problem solving with the amount of alcohol ingestion.

Neural hyperconnectivity is characterized by an abnormal neural linking of brain areas that are not typically functionally connected. Hyperconnectivity is linked to bizarre associations in schizophrenia (Whitfield-Gabrieli et al., 2009), and has also been noted in bipolar individuals (McCrea, 2008). Brain imaging studies detect more alpha synchronization, both within and across hemispheres, in the brains of high creative vs. less creative subjects, suggesting, perhaps, unusual connectivity (Fink and Benedek, 2014). The exploration of neural connectivity in creative but schizotypal individuals may shed light on the creation of remote associations.

Mood lability is a characteristic of mood disorders. Changes in mood, especially increases in positive affect, have been shown to increase divergent thinking in normal subjects (Ashby et al., 1999), while highly creative individuals with mood disorders demonstrate patterns of higher creative productivity during upswings in mood (Jamison, 1989). However, the neuroscience of creativity and mood is not well-explored and presents a ripe area for further inquiry.

## Conclusions

The mad genius debate is a polarizing and divisive issue in the field of creativity research. By reframing this debate as a question of how individuals with vulnerability to psychopathology differ in their strategies of solving creative tasks from those who do not display evidence of vulnerabilities, we can use the mad genius issue as an opportunity to promote a scientific exploration of creativity and psychopathology rather than to polarize the field.

The implications of the mad genius debate for the neuroscience of creativity are threefold. First, the debate emphasizes the need for, and encourages, an individual difference methodology rather than a universal one-size-fits-all approach to the neuroimaging of creative tasks. Individual differences related to psychopathology, including self-report measures of schizotypy, hypomania, alcohol use, and creative achievement, can easily be added to research protocols, and may help explain conflicting findings in imaging studies.

Second, studies that target shared vulnerabilities related to creativity and psychopathology, as well as non-shared risk and protective factors, can illuminate the neural underpinnings of creative cognition that appear to allow some individuals at risk for psychopathology to have a creative edge. Clinicians who treat creative populations cite high percentages of non-compliance with drug treatment because of the negative effects of treatment on creativity (Flaherty, 2011). Imaging studies may aid in determining which symptoms of psychopathology are creativity-enhancing and, thus, suggest directions for the development of symptom-specific drug and psychological therapies that will leave creativity in tact while improving quality of life in those with associated psychopathology.

Finally, a neuroscience of creativity and psychopathology may reveal novel strategies of summoning the muse that may then be employed to assist non-disordered individuals in enhancing their creativity, thus enriching both their own lives and society as a whole.

### Conflict of interest statement

The author declares that the research was conducted in the absence of any commercial or financial relationships that could be construed as a potential conflict of interest.
